# Pollution and Oral Bioaccessibility of Pb in Soils of Villages and Cities with a Long Habitation History

**DOI:** 10.3390/ijerph13020221

**Published:** 2016-02-17

**Authors:** Nikolaj Walraven, Martine Bakker, Bertil van Os, Gerard Klaver, Jack Jacobus Middelburg, Gareth Davies

**Affiliations:** 1 GeoConnect, Meester Dekkerstraat 4, Castricum 1901 PV, The Netherlands; 2 Rijksinstituut voor Volksgezondheid en Milieu (RIVM), P.O. Box 1, Bilthoven 3720 BA, The Netherlands; martine.bakker@rivm.nl; 3 Rijksdienst voor het Cultureel Erfgoed, Cultural Heritage Agency, P.O. Box 1600, Amersfoort 3800 BP, The Netherlands; B.van.Os@cultureelerfgoed.nl; 4 Bureau de Recherches Géologiques et Minières (BRGM), 3 Avenue Claude-Guillemin, BP 36009, Orléans Cedex 2 45060, The Netherlands; gerardklaver3@gmail.com; 5 Department of Earth Sciences, University Utrecht, P.O. Box 80021, Utrecht 3508 TA, The Netherlands; j.b.m.middelburg@uu.nl; 6 Geology & Geochemistry, Faculty of Earth and Life Sciences, VU University Amsterdam, De Boelelaan 1085, Amsterdam 1081 HV, The Netherlands; g.r.davies@vu.nl

**Keywords:** lead, pollution, soil, isotopes, sources, oral, bioaccessibility

## Abstract

The Dutch cities Utrecht and Wijk bij Duurstede were founded by the Romans around 50 B.C. and the village Fijnaart and Graft-De Rijp around 1600 A.D. The soils of these villages are polluted with Pb (up to ~5000 mg/kg). Lead isotope ratios were used to trace the sources of Pb pollution in the urban soils. In ~75% of the urban soils the source of the Pb pollution was a mixture of glazed potsherd, sherds of glazed roof tiles, building remnants (Pb sheets), metal slag, Pb-based paint flakes and coal ashes. These anthropogenic Pb sources most likely entered the urban soils due to historical smelting activities, renovation and demolition of houses, disposal of coal ashes and raising and fertilization of land with city waste. Since many houses still contain Pb-based building materials, careless renovation or demolition can cause new or more extensive Pb pollution in urban soils. In ~25% of the studied urban topsoils, Pb isotope compositions suggest Pb pollution was caused by incinerator ash and/or gasoline Pb suggesting atmospheric deposition as the major source. The bioaccessible Pb fraction of 14 selected urban soils was determined with an *in vitro* test and varied from 16% to 82% of total Pb. The bioaccessibility appears related to the chemical composition and grain size of the primary Pb phases and pollution age. Risk assessment based on the *in vitro* test results imply that risk to children may be underestimated in ~90% of the studied sample sites (13 out of 14).

## 1. Introduction

Pollution of soils with heavy metals, including lead (Pb), started with the domestication of fire [1]. The remnants of burnt firewood are rich in heavy metals and alter the metal content of the topsoil near the fireplace. Lead pollution became significant after the discovery of cupellation (extraction of silver from lead ores) around 3500 B.C [2] and reached its peak in the 1970s when leaded gasoline was the major energy source for cars world-wide [3,4,5,6]. Adoption of a series of regulations to ban leaded gasoline in the 1970s, drastically reduced Pb emissions to the environment [7,8,9,10,11].

Lead pollution in soils is generally recognised by elevated contents of Pb and/or other heavy metals in the topsoil. Since Pb accumulation in soils can also be the result of natural processes, Pb isotope measurements are an effective tracer to establish whether excess Pb contents are from anthropogenic Pb sources [12,13,14,15,16,17,18,19]. Concerns regarding the possible human- and ecotoxicological risks of soils polluted with Pb has led to numerous studies of Pb pollution in various parts of the world since the 1970s [12,13,20,21,22,23]. The majority of these studies focussed on the atmospheric Pb input to soils and sediments [7,8,24,25,26,27]. Very few studies tried to trace and quantify the input of anthropogenic Pb to soils related to domestic activities. Notable exceptions are Walraven *et al.* [14] and Hansmann and Köppel [15].

The ubiquity of Pb in and around the household, even in Roman times, is well-known [28]. In addition to the often cited lead-paint chips and house dust, high Pb contents have also been found in printed matter, wrapping paper, textiles, ceramics (pots and plates), constructing materials (lead slabs) and even toothpaste [2]. Some of these household and construction artefacts enter soils where they may constitute a highly localised source of Pb pollution. Lead in house paints, for example, can be transferred to soil by natural weathering or by burning-off, sanding or scraping of the old paint before repainting. High Pb contents (up to 1.7 wt.%; [29]) have been found in soils and dirt in the immediate vicinity of old wooden houses [14,29,30].

Although household and construction artefacts only cause very localised Pb pollution, it may pose a threat to human health. Remnants of Pb-containing artefacts may end up in the topsoil of gardens where people grow vegetables and children play. Lead can enter the human body after oral ingestion of vegetables and soil, either accidentally via hand-to-mouth behaviour or deliberately. Children are far more sensitive to Pb poisoning than adults [31]. Even at low exposure levels, Pb causes impairment of normal neurological development in children leading to learning and reasoning difficulties, retardation of physical development, hearing loss, hyperactivity, and reduced attention span [4]. Effects in adults include elevated blood pressure and hypertension, resulting in increased risk of cardiovascular diseases, and renal deficiencies.

During the last decade many *in vitro* tests have been developed and evaluated to estimate the oral bioavailability of contaminants/compounds, including Pb, in soils [32]. With these tests, oral Pb bioaccessibility can be determined as an indication for the maximum oral Pb bioavailability of Pb polluted soils. There is evidence that the oral bioavailability of Pb in soils depends on the type of anthropogenic Pb source present. Walraven *et al.* [32], for example, have shown that the oral Pb bioaccessibility, based on the RIVM *in vitro* test [33], decreased in the following order: Pb bullets and pellets > Car battery Pb > Made ground Pb ≈ Gasoline Pb ≈ Diffuse Pb > City waste (also known as municipal solid waste).

Soils from villages and cities with a long habitation history usually contain various anthropogenic Pb sources and the specific make of these sources will influence the bioavailability, and related human risks, to a great extent. The aim of this study is to determine the sources, mainly related to domestic activities, and oral accessibility of Pb pollution in soils of two villages and two cities with a long habitation history. The two cities were already inhabited in Roman times and the two villages were founded around 1600 A.D. Lead isotope analysis is used to trace the anthropogenic Pb sources responsible for Pb pollution in soils covering the Roman (57 B.C.–350 A.D.), Medieval (500 A.D.–1500 A.D.) and Modern (1500 A.D.–2003 A.D.) time periods.

## 2. Background Information

A distinction between natural and anthropogenic Pb, and between the anthropogenic Pb sources can only be made if the Pb isotope composition of the various Pb sources differs significantly. Walraven *et al.* [14] demonstrated that with a combination of Pb isotope analysis, major and trace element analysis and fuzzy clustering, natural Pb in soils can be distinguished from anthropogenic Pb. In addition, three sources of Pb pollution in soils could be distinguished: (1) construction materials of old houses (building materials such as Pb sheets, glazed roof tiles and paint); (2) coal ashes and (3) alkyl-leaded petrol. The village of Graft-De Rijp was used as the basis of the Walraven *et al.* [14] study. In the present study we extended the research on Pb pollution due to domestic activities, from one village/city to four. Furthermore, the habitation history of the newly investigated cities/villages is longer: from 57 BC to 2003 A.D. instead of 1612 A.D. to 1997 A.D. With a greater number of soil samples and Pb containing artefacts, we attempted to resolve more sources of Pb pollution and to better quantify specific Pb polluting activities.

**Figure 1:**
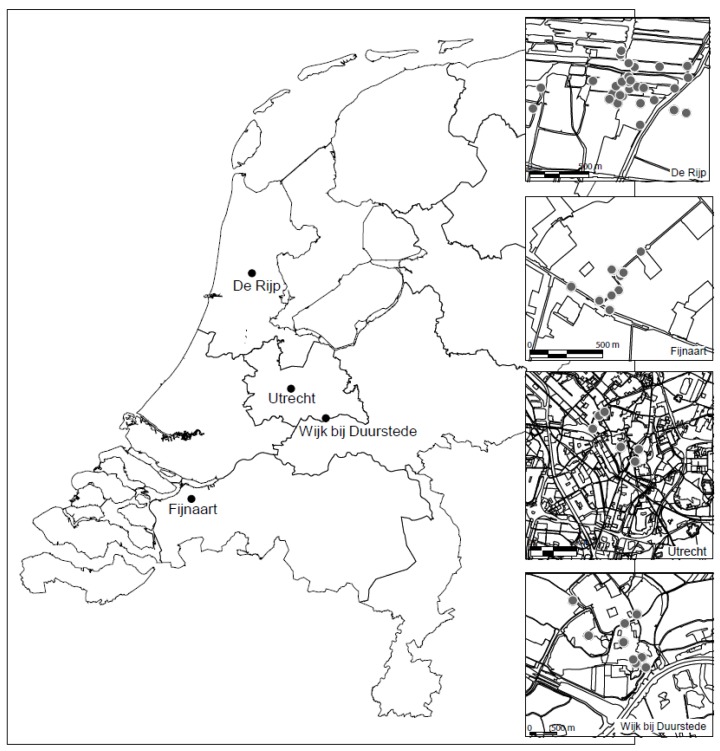
Sample locations of the Pb polluted soils and potential anthropogenic Pb sources.

The 4 studied villages/cities—in order of habitation—are Utrecht, Wijk bij Duurstede, Fijnaart and Graft-De Rijp. The approximate locations of these cities and villages are shown in Figure 1, and their precise location and date of sample collection in Table 1. Various environmental studies have shown that the soils in all 4 villages/cities are polluted with Pb and related heavy metals [14,34,35].

### 2.1. Utrecht

Utrecht is located approximately 40 km southeast of Amsterdam (Figure 1). It is the fourth largest city in The Netherlands, with a population of ~316,000 persons [36]. The high sandy river banks of the Crooked Rhine and the Old Rhine—where Utrecht is now located—were already inhibited during the Bronze Age (1800–800 B.C.) [37]). In 47 A.D. the Romans arrived and built an outpost on the south bank of the Crooked Rhine on a fordable point which was called *Trajectum* *ad Rhenum (*Lat. = fort of the Rhine) [37]. Utrecht was one of the forts on the northern borders of the Roman Empire intended to ward off invasions from Germania. Many Roman artefacts are found in Utrecht and its surroundings, among others lead weights, leaden coins, leaden ornaments and leaden eating utensils [38].

Around 275 A.D. the Romans left Utrecht and little is known about the period 275 to 650 A.D. [37]. During the Middle Ages (500 to 1500 A.D.) Utrecht was a religious and commercial centre [39]. Due to trading activities, Utrecht became a prosperous city with renowned annual fairs. Trade and industry was accompanied by intensive shipping activities, resulting in the construction of many canals and wharfs [39]. In this time period the use of lead for painting, roofing materials, manufacturing of glass and Pb glazed pottery increased [31]. In addition the ubiquitous pewter ware contained high Pb content during these times [31].

With the onset of the Industrial Revolution some small scale Pb working industries were established in Utrecht (e.g., Pb white factory and Pb flatting mills) [40]. Industrialisation, however, only gained momentum in and around Utrecht late in the 19th century and today the local economy predominantly is based mainly on service activities.

### 2.2. Wijk bij Duurstede 

Wijk bij Duurstede is located close to Utrecht (20 km southeast of Utrecht; Figure 1) and has a population of ~23,000 persons [36]. Its history resembles that of Utrecht with evidence for habitation in the Bronze age [41]. In 57 B.C. the Romans settled here and built a fort, presumably called Levefanum, to protect themselves from invasions from Germania [41]. In the early middle ages, a settlement named Dorestad emerged at the site of the Roman fortress. Dorestad was an important trade settlement that drew the attention of the Vikings, who frequently raided the settlement in the 9th century [41]. Wijk bij Duurstede is a small city with no significant industries on the site of Dorestad.

### 2.3. Fijnaart

Fijnaart is located ~90 km south of Amsterdam (Figure 1) and has a population of ~5,000 persons [36]. Little information is known about the history of Fijnaart. It was founded in 1547 A.D. in a polder [42]. The main buildings were dike houses and ribbon settlement. The main economic activities are agriculture and fishery. There is no significant industry.

### 2.4. Graft-De Rijp

Graft-De Rijp is located ~25 km north of Amsterdam and has a population of ~6000 persons [36]. A summary of the history of Graft-De Rijp is given below, taken from Walraven *et al.* [14]. In 1612 A.D., the Beemster (area surrounding Graft-De Rijp) was reclaimed from the sea. Around this time De Rijp was founded. Until the 17th century houses in this village were entirely constructed from wood, which was treated with Pb-based paint and roofed with glazed (Pb-based) and unglazed tiles. During this period rain pipes and gutters were predominantly made from Pb and Zn. In the mid-17th century an economic boom (as a result of whaling) stimulated the development of sites for ship building, sail-lofts and industrial mills. Waste products of these activities were used to raise the land. During this time, three large fires destroyed many houses (in 1654 A.D., 1657 A.D. and 1674 A.D.). The debris from the fires was used to raise more of the land. In the 18th century an economic recession took place. From this time until the 19th century many buildings became derelict or were destroyed. The waste products were again used to raise the land and to fill ditches and channels. In the industrial era (after 1860 year A.D.) gasworks, coal storehouses, printing-works and tanneries were started. Subsequently, the town centre was rebuilt on a mixture of sludge, manure and town refuse [14].

## 3. Methods

### 3.1. Site Selection and Soil Sampling

Between January 1996 and May 2003 a total of 137 soil samples were collected from 79 sample sites in four villages/cities in The Netherlands. Soil samples were obtained by driving an Edelman drill up to a depth of 2 m. All cores were described and at least one sample was taken from each soil horizon.

The selected sample sites have been very well studied and documented by archaeologists [41,43,44]. Archaeologists accompanied us in the field when sampling soils. Based on previous archaeological studies and direct field observations by the archaeologists, chronology (Roman, Medieval or Modern time period) was assigned to the soil samples.

These selected sites represent both unpolluted areas and those suspected of local pollution. The unpolluted sites are situated in the open field or at depths >1 m, where no anthropogenic influence could be detected visually. In contrast, the presumed polluted sites were chosen on the basis of findings by environmental contractors of high Pb, Zn and Cu contents.

A total of 75 possible Pb sources (artefacts) were collected in the field and by archaeologists working in the study area. These artefacts include Pb sheets, ceramics and paint. The production period of the artefacts was determined by archaeologists and based on historical knowledge of the sample sites, but it was not always possible to appoint an exact date. We were, however, able to determine the archaeological time period in which the artefacts were made and used. Some of these artefacts can be dated back to Roman times. Other possible Pb sources are coal and coal ashes. Since coal is dispersed through the soils, pure coal and coal ashes samples could not be collected. Therefore, we used twenty coal samples from Dutch and Belgian coalmines (coal data reported in Walraven *et al.* [14,19]. Approximately 90% of all coal used for domestic purposes comes from Dutch and Belgian coal mines [45].

Trace element contents and Pb isotope ratios of the Graft-De Rijp soils and potential Pb sources were originally reported by Walraven *et al.* [14]. In the present work Pb isotope data are placed in a broader context—more cities/villages and more time periods—and therefore these data are again reported and discussed.

In August–September 2007, eight sample sites in Wijk bij Duurstede, Utrecht and Graft-De Rijp were revisited by RIVM and 14 Pb polluted soil samples were taken for the *in vitro* Pb bioaccessibility tests [34]. The Pb polluted soils in Fijnaart were already remediated and were therefore not sampled again. Only soils with Pb contents higher than the current Dutch intervention value for Pb (530 mg/kg for standard soils; 25% clay and 10% organic matter) were sampled. Lead contents were determined in the field using a NITON Xl3t handheld XRF. Since soil Pb content had to be higher than 530 mg/kg, only soils polluted in the Modern period were sampled. In Wijk bij Duurstede and Graft-De Rijp soil inferred to be polluted with city waste were sampled. In Utrecht sampled soils are inferred to be polluted with city waste or Pb white (in the vicinity of a former Pb white factory). For further details, see Hagens *et al.* [34].

### 3.2. Sample Preparation

Prior to analysis, soil samples and artefacts from Graft-De Rijp were dried at 60 °C to constant weight. All other samples were dried at 105 °C to constant weight. The dried samples were ground (<20 um) with an automated tungsten-carbide mill (Herzog HSM-HTP), homogenised for 5 min in a Turbula T2C and stored in glass containers. Some samples could not be ground with the automated tungsten-carbide mill (among others painted wood and Pb sheets). These samples were ground manually with an agate mortar or not ground at all.

### 3.3. Analytical Procedure

The soil samples and the majority (96%) of the artefacts were analysed for Pb and Al content using X-ray fluorescence (XRF) spectrometry. Aluminium (Al) and lead (Pb) have been measured by XRF with a precision (1 RSD)—based on replicate analysis—of 1.6% and 4.8% respectively. For details see Van der Veer [46] and Walraven *et al.* [47]. One reference sample (ISE 921) was added to each batch of 20 samples to determine accuracy. The certified Al and Pb content of ISE 921 is 5.7 wt.% and 167 mg/kg respectively. The accuracy for Al and Pb is 14% and 1.9% relative bias respectively.

Some artefacts (4%), all from Graft-De Rijp, could not be pressed into tablets (e.g., Pb sheets and paint on wood). After HF based digestion the Pb content of these samples was analysed with a VG Plasmaquad PQ2+ Inductively Coupled Plasma Mass Spectrometer (ICP-MS) with a low uptake nebulizer. Details of the ICP-MS method can be found in Huisman *et al.* [48]. The relative precision (2RSD) and accuracy for Pb was 11.3% and 0.005% respectively (relative bias).

Lead isotopes were analysed after HF-based sample destruction. Details of the HF-based destruction methods can be found in Walraven *et al.* [14] and Van der Veer [46] for the Graft-De Rijp and other samples respectively. Lead isotope compositions of the Graft-De Rijp samples were determined with a VG Plasmaquad PQ2+ ICP-MS. Details of this method are described by Walraven *et al.* [14]. Lead isotopes of all other samples were analysed with an Agilent 7500 ICP-MS. A similar method to Krachler *et al.* [49] was adopted to correct for mass bias discrimination by bracketing each six samples with the Pb isotope standard NIST 981. The effect of count rate on the mass discrimination was minimized by diluting all residues and isotope standards to a lead concentration of 50 ug/kg. Details of this method are described by Walraven *et al.* [19,47]. All isotopic ratios of the Graft-De Rijp samples were determined with a precision of <1%, 2RSD. The precision (2RSD) of all other samples was <0.29% for ^206^Pb^/207^Pb, <0.24% for ^208^Pb/^207^Pb, and <0.55% for ^206^Pb/^208^Pb. The average Pb isotope composition and precision (2 SD) of the measured ISE 921 sample was 1.166 ± 0.003 for ^206^Pb/^207^Pb, 2.444 ± 0.004 for ^208^Pb/^207^Pb, and 0.477 ± 0.002 for ^206^Pb/^208^Pb. Average precision and accuracy are based on the entire analytical procedure starting with the sample splits. Blanks indicate reagents contain negligible amounts of Pb (<20 ng/kg).

The RIVM *in vitro* model, introduced by Oomen *et al.* [33] was used to determine the oral bioaccessibility of Pb in Pb polluted soil samples. In the *in vitro* gastrointestinal model dried and sieved soil samples (fraction < 2 mm) were subjected to a number of stages simulating the human digestion process (under fasted conditions; worst case scenario). Duplicate pre-treated subsamples (dried, sieved, not crushed) of 0.06 g were weighed into centrifuge tubes and 9.0 mL of saliva (pH 6.5 ± 0.2) was added. This mixture was rotated for 5 min, end-over-end, at 2900 g (about 55 rpm) at 37 °C. Then, 13.5 mL of gastric juice (pH 1.1 ± 0.1) was added, and the mixture was rotated for 2 h at 37 °C. The pH of the mixture was measured to check that solutions were within method specified pH tolerance of 1.5 ± −0.5. Finally, 27 mL of duodenal juice (pH 7.8 ± 0.2) and 9 mL of bile juice (pH 8.0 ± 0.2) were added simultaneously. This mixture was rotated at 37 ºC for 2 h and subsequently centrifuged at 3000 *g* for 5 min. This yielded the chyme (the supernatant; gastric + intestinal fraction) and the pellet (the residual soil). The Pb content of the 14 soil samples (and the residual pellets) was determined with ICP-MS according NEN-ISO 17294 [50] after microwave assisted destruction according to NEN 6961 [51], except that a 1:3 dilution of aqua regia with distilled water was used. The Pb content in the chyme samples was determined according to NEN-ISO 17294 [50] after dilution in 0.1 M HNO_3_.

The RIVM *in vitro* method used in this study differs slightly from the RIVM *in vitro* method used by Walraven *et al.* [32]. Instead of 0.6 g, 0.06 g of soil sample was introduced in the model. RIVM decided to decrease the solid to liquid ratio (from 1:100 to 1:1000) because the oral exposure of young children to soil, due to hand-to-mouth behaviour, is lower (0.06 g instead of 0.6 g) than previously thought [34]. The smaller solid to liquid ratio (1:1000) results in higher oral bioaccessibilities of Pb from soils [34]. For further details see Van de Wiele *et al.* [52] and Hagens *et al.* [34].

### 3.4. Data Analysis

Polluted soils contain both natural and anthropogenic Pb. Consequently, the measured Pb isotope composition is a mixture of the isotopic composition of both the natural and anthropogenic Pb fraction. To derive the Pb isotope composition of the anthropogenic Pb fraction, it is necessary to account for the amount of natural Pb present in the soils. The following equation describes this mass balance principle:

(^x^Pb/^y^Pb)_a_ × Pb_a_ = (^x^Pb/^y^Pb)_t_ × Pb_t_ − (^x^Pb/^y^Pb)_n_ × Pb_n_ (1)

In which the letters a, t and n indicate the Pb content and Pb isotope composition (^x^Pb/^y^Pb) of the **a**nthropogenic, **t**otal and **n**atural Pb fraction, respectively. (^x^Pb/^y^Pb)_t_ and Pb_t_ of the soils are measured in this study. The natural Pb content (Pb_n_) in sediments and soils can be calculated based on the common relationship between Pb and Al in unpolluted soils [6,46,47,53] and, because Dutch soils are seldom polluted with Al:

Pb_n_ = 3.69 × Al + 1.75 (2)

In which Pb_n_ is the calculated natural Pb content in mg/kg, and Al is the measured Al content in wt.% (*n* = 303; R^2^ = 0.89; Standard error of estimate is 2.6 mg/kg).

The anthropogenic Pb content in the topsoils Pb_a_ can then be calculated as follows:

Pb_a_ = Pb_t_ − Pb_n_ (3)

In which Pb_a_ and Pb_n_ (Equation (3)) are the calculated anthropogenic and natural Pb content in mg/kg in the soils, and Pb_t_ is the measured total Pb content in mg/kg in soils.

If the Pb isotope composition of the natural Pb fraction in the soils is known, the Pb isotope composition of the anthropogenic Pb fraction can be calculated using Equation (1). Potentially the Pb isotope composition of the natural Pb fraction in soils can be estimated by analysing the unpolluted deeper soils (>1 m). Unfortunately, the majority (60%) of the deeper soils appears to be polluted with anthropogenic Pb (see Supplementary 1). The variation in Pb isotope composition of background Pb in Dutch topsoils was determined by Walraven *et al.* [47] based on natural sand(*n* = 184), fluviatile (*n* = 22) and marine clay (*n* = 81).The median values of these data are used for the correction. If the anthropogenic Pb content in soils is low compared with the natural Pb content, the error in the calculated Pb isotope composition of the anthropogenic Pb fraction can be substantial. To minimize this error, (^x^Pb/^y^Pb)_a_ is calculated only for samples for which Pb_t_/Pb_n_ > 2 (Pb enrichment factor (called EF)).

Provided the sum of the Pb content in the chyme and the Pb content in the residual pellet does not differ significantly from the total Pb content of the soil sample, the bioaccessibility of Pb can be calculated according to Equation (4):

F_B-Pb_ = (Pb_chyme_/Pb_t_) × 100% (4)

In which F_B-Pb_ is the calculated bioaccessibility of Pb (%), Pb_chyme_ is the measured Pb content determined in chyme (mg/kg) and Pb_t_ is the measured total Pb content in the soil samples.

## 4. Results

### 4.1. Chemical and Pb Isotope Composition of the Soil Samples

**Figure 2:**
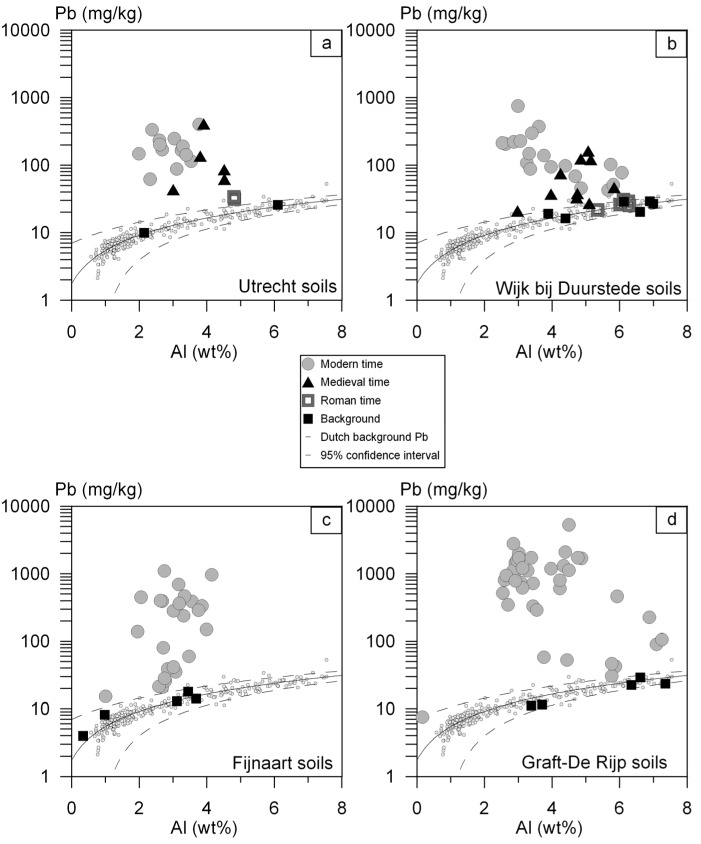
Pb content (mg/kg) of the soil samples—background or influenced in the Roman, Medieval or Modern time—*versus* Al content (wt.%) per city/village. The line indicates the average relationship between the Al and Pb content of unpolluted Dutch sedimentary soils, based on 303 samples (●) [47]. The dashed lines are the 95% confidence intervals of the relationship between Al and Pb. **a** = Utrecht; **b** = Wijk bij Duurstede; **c** = Fijnaart; **d** = Graft-De Rijp.

**Figure 3:**
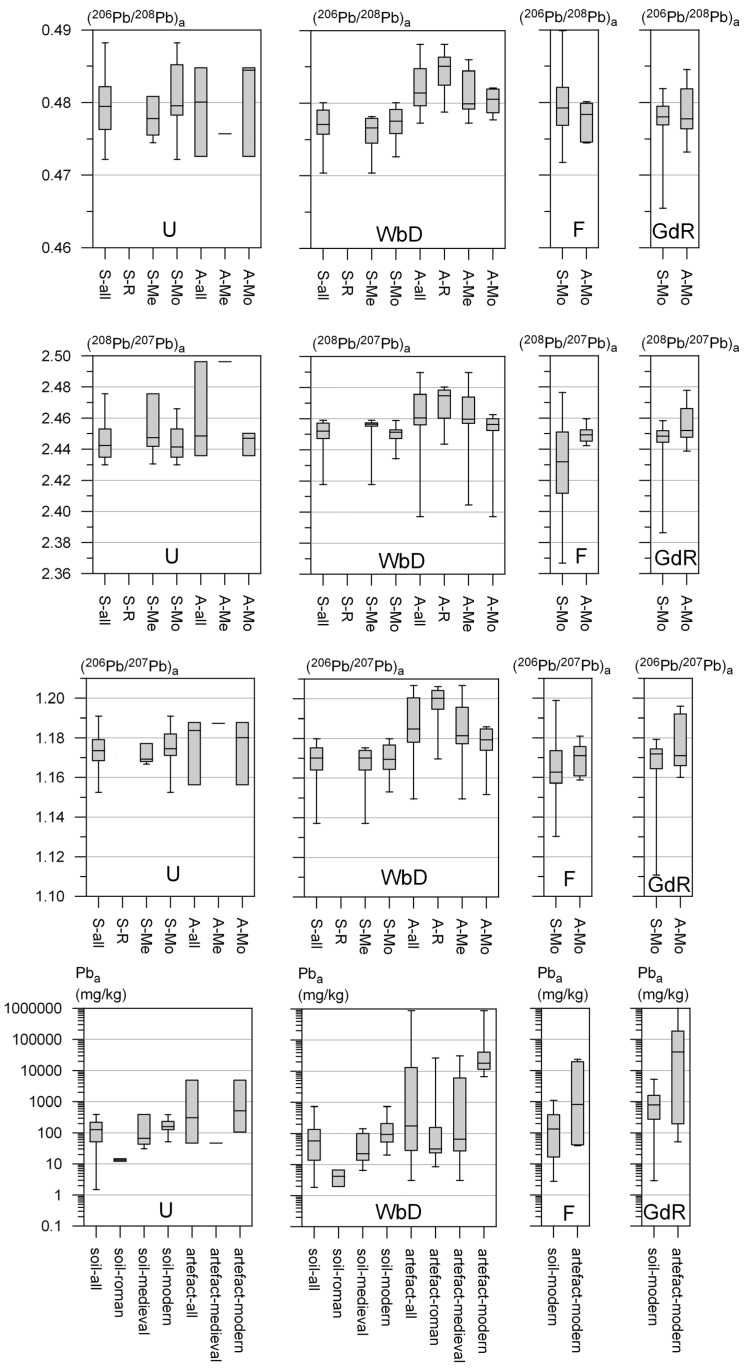
Box-whisker plots of Pb_a_, (^206^Pb/^207^Pb)_a_, (^208^Pb/^207^Pb)_a_ and (^206^Pb/^208^Pb)_a_ in the urban soils (S) and potential Pb artefacts (A) showing the minimum (lower whisker), maximum (upper whisker), median (centre of the box), lower quartile (bottom of box), and upper quartile (top of box) values. U = Utrecht; WbD = Wijk bij Duurstede; F = Fijnaart; GdR = Graft-De Rijp.

Sample details (including lithology, sample site coordinates and sample depth), analytical and calculated results of the soil samples are given in Supplementary 1. Table 2 summarizes the measured Al content and the calculated Pb and Pb isotope composition of the anthropogenic Pb fraction of all soil samples categorized per city/village (Utrecht, Wijk bij Duurstede, Fijnaart and Graft-De Rijp) for each time period (Roman, Medieval, Modern). In Figure 2a–d the total Pb contents of the soil samples are plotted *versus* the Al contents. Eighteen deeper soil samples (>1 m), indicated with ■, have Pb contents matching those of natural Pb (Figure 2a–d). All other soil samples (*n* = 119) were visually disturbed and/or are enriched in Pb (Supplementary 1, Figure 2a–d). The variation in anthropogenic Pb content and its isotopic composition are presented in box-whisker plots in Figure 3 for the four cities/villages and the three time periods. Pb_a_ varies between < limit of detection (LOD) and 5266 mg/kg.

The median soil Pb_a_ content in the four cities/villages increases in the following order (Table 2): Wijk bij Duurstede (57 mg/kg) < Utrecht (127 mg/kg) < Fijnaart (133 mg/kg) < Graft-De Rijp (789 mg/kg). The median Pb_a_ content for the soils from the three distinguished time periods increases from Roman period (7 mg/kg) to Medieval period (43 mg/kg) to Modern period (235 mg/kg) (Table 2). Only two of the seven soil samples that contain visually recognisable remains from Roman times have enriched Pb contents (Supplementary 1, Figure 2a). These two samples are from Utrecht (Figure 2a). All samples that contain visually recognisable remains from the Medieval or Modern period have enriched Pb contents (Supplementary 1).

The Pb isotope composition of the anthropogenic Pb fractions is calculated only for the soils with EF > 2 (see Section 3.4.). This is the case for ~75% of all soils (102 out of 137). The (^206^Pb/^207^Pb)_a_ ratios vary between 1.111 and 1.199 with a median of 1.171 (*n* = 102; Table 2, Figure 3). Excluding the lowest and highest values (*n* = 100), the range is reduced to between 1.130 and 1.195 (Supplementary 1). Soils from Utrecht, Wijk bij Duurstede and Graft-De Rijp have comparable median (^206^Pb/^207^Pb)_a_ ratios of 1.174, 1.170 and 1.172 respectively (Table 2, Figure 3). The soils from Fijnaart have a slightly deviating median (^206^Pb/^207^Pb)_a_ ratio of 1.163 (Table 2, Figure 3). This—lithology independent—regional difference in (^206^Pb/^207^Pb)_a_ ratio is observed for all the time periods (Table 2, Figure 3).

The (^208^Pb/^207^Pb)_a_ and (^206^Pb/^208^Pb)_a_ ratios vary between 2.367 and 2.476 with a median of 2.447, and between 0.465 and 0.490 with a median of 0.478, respectively (*n* = 102; Table 2 and Figure 3). If the soil samples with the single lowest and highest ratio are disregarded, the ratios vary between 2.386 and 2.475, and between 0.470 and 0.490 (*n* = 100). Similar patterns are observed for the (^206^Pb/^207^Pb)_a_, (^208^Pb/^207^Pb)_a_ and (^206^Pb/^208^Pb)_a_ ratios.

### 4.2. Potential Anthropogenic Pb Sources (Pb Artefacts)

Sample details (including sample location and time period) and analytical results of the potential anthropogenic Pb sources are given in Table 3. The variation in Pb content and isotopic composition is presented in box-whisker plots in Figure 3 for the four cities/villages and the three time periods.

The Pb content of the potential Pb sources varies from 3 mg/kg (Medieval production slag) to ~100 wt.% (Modern Pb sheet). High Pb contents are measured in glazed potsherds (0.5–8.7 wt.%), glazed roof tiles (1.9–5.9 wt.%), paint (6.0–18.6 wt.%), Pb spool (87.8 wt.%) and Pb sheets (~100 wt.%) (Table 3). Low Pb contents are measured in unglazed sherds and potsherds (e.g., 11–40 mg/kg in Roman potsherds) (Table 3). The Pb content of the unglazed sherds and potsherds matches that of clayey soils in The Netherlands [47]. Metal and production slags contain variable Pb contents (e.g., 3–29 mg/kg in Medieval production slag from Wijk bij Duurstede and 58–2265 mg/kg in Medieval production remnants from another site in Wijk bij Duurstede) (Table 3). The Pb content of the potential anthropogenic Pb sources from the 3 distinguished time periods increases in the following order (Table 3, Figure 3): Roman period < Medieval period < Modern period. The artefacts with a Pb content lower than or equal to the background soil Pb content (e.g., unglazed potsherds) cannot cause Pb pollution and are therefore not considered as anthropogenic Pb sources in the discussion.

The ^206^Pb/^207^Pb, ^208^Pb/^207^Pb and ^206^b/^208^Pb ratios of the potential Pb sources vary between 1.150–1.207, 2.397–2.496 and 0.473–0.488 respectively (Table 3). Lead containing artefacts with low Pb contents (<100 mg/kg) are, in general, characterized by high isotopic ratios (Table 3). For example, ^206^Pb/^207^Pb, ^208^Pb/^207^Pb and ^206^b/^208^Pb ratios of unglazed Roman potsherds range between 1.195–1.206, 2.472–2.480 and 0.483–0.487 respectively (Table 3). These ratios match that of unpolluted clays in The Netherlands [47]. Lead containing artefacts with the highest Pb contents are, in general, characterized by low isotopic ratios. For example, ^206^Pb/^207^Pb, ^208^Pb/^207^Pb and ^206^b/^208^Pb ratios of glazed potsherds and roof tiles range between 1.152–1.188, 2.397–2.460 and 0.478–0.485 respectively (Table 3).

### 4.3. In Vitro Digestion Model

The Pb content of the soil, chyme and residual pellets, and the calculated Pb bioaccessibilities (Equation (4)) are listed in Table 4. In addition, Pb isotope composition (if available) and inferred Pb sources are presented. The Pb content of the soil and chyme samples varies from 540 to 2335 mg/kg and 175 to 1355 mg/kg respectively (Table 4). The (^206^Pb/^207^Pb)_t_ and (^208^Pb/^207^Pb)_t_ values of the soil samples vary from 1.156 to 1.179 and from 2.437 to 2.456 respectively (Table 4).

The Pb isotope composition of the total amount of Pb in this set of soil samples is assumed to be equal to the anthropogenic Pb fraction since the natural Pb content is negligible compared to the total Pb content (2–29 mg/kg *versus* 540–2335 mg/kg).

Since the sum of Pb present in the chyme and the residual pellet does not differ significantly from the total Pb content present in the soil samples bioaccessibility can be calculated quantitatively according to Equation (4) (log(Pb_t_) = 1.03 ± 0.03 × log(Pb_chyme_ + Pb_pellet_); through origin; R^2^ = 0.91; *p* < 0.05). Although log(Pb_t_) does not differ significantly from log(Pb_chyme_ + Pb_pellet_), the difference between the measured total Pb content and the sum of the Pb content in the chyme and in the residual pellet can be substantial (Table 4). This is caused by the presence of heterogeneously distributed, coarse Pb containing particles in the soil samples. Since the soil samples were not ground for the *in vitro* test (this reflects hand-to-mouth behaviour of children best), the presence or absence of such particles in a sample can influence the Pb content. Calculated (Equation (4)) bioaccessibilities range from 16% (soil polluted with city waste in Graft-De Rijp) to 82% (soil polluted with Pb white from Utrecht).

## 5. Discussion

### 5.1. Anthropogenic Pb Sources in Urban Soils

**Figure 4:**
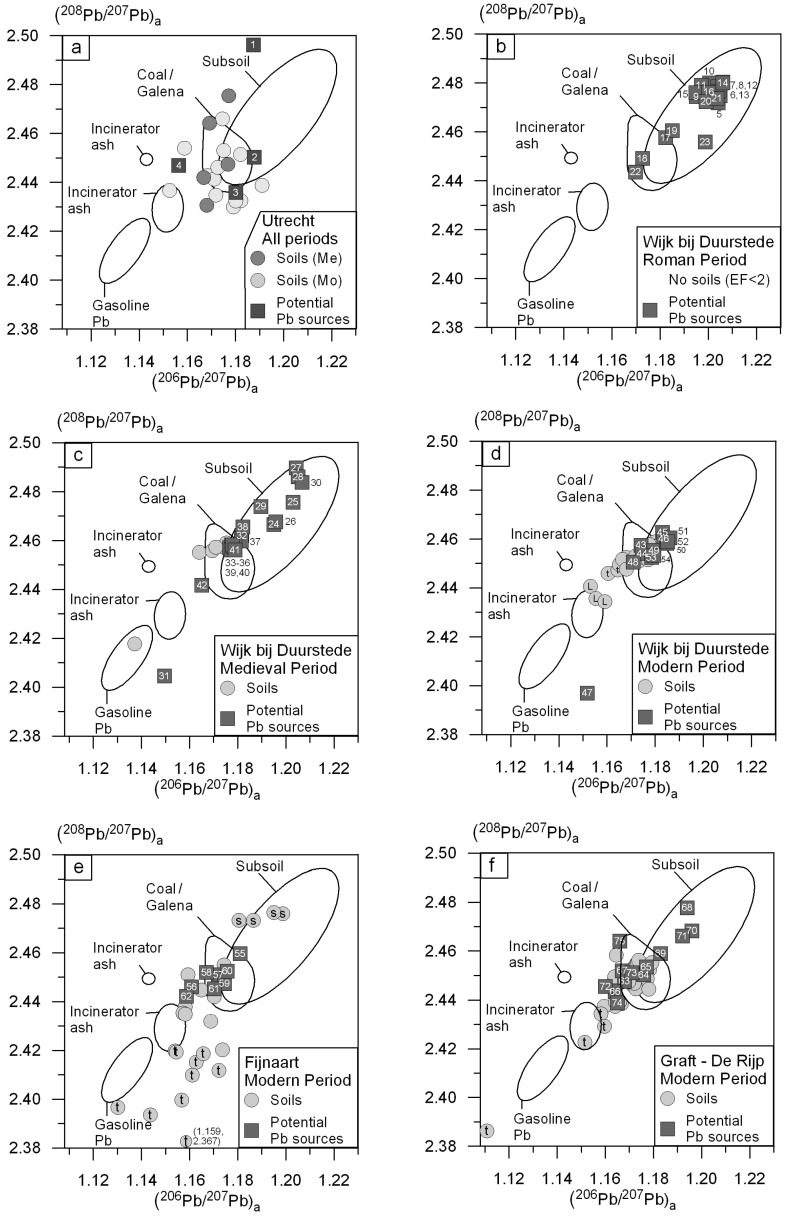
(^208^Pb/^207^Pb)_a_ versus (^206^Pb/^207^Pb)_a_ in urban soils and potential anthropogenic Pb sources in The Netherlands. Ellipses represent the lithologically inherited Pb isotope composition of Dutch soils and the Pb isotope composition of potential anthropogenic Pb sources (gasoline Pb, incinerator ash, coal and galena ore). References ellipses: Subsoil: Walraven *et al.* [47]. Coal: Walraven *et al.* [14,19]. Galena: Pasteels *et al.* [54] and Cauet *et al.* [55]. Gasoline Pb: Walraven *et al.* [6]. Incinerator ash: Monna *et al.* [56], Chiaradia and Cupelin [57], Hansmann and Köppel [15], Carignan *et al.* [58] and Cloquet *et al.* [16]. L = landfill, t = topsoil, S = subsoil, R = Roman, Me = Medieval, Mo = Modern, **a** = Utrecht (all periods); **b** = Wijk bij Duurstede (Roman period); **c** = Wijk bij Duurstede (Medieval period); **d** = Wijk bij Duurstede (Modern period); **e** = Fijnaart (Modern Period); **f** = Graft-De Rijp (Modern period). Numbers in the rectangles match with the artefact numbers in Table 3.

To trace the sources of anthropogenic Pb in the polluted soils of four Dutch cities/villages with a long habitation history, (^208^Pb/^207^Pb)_a_ of the soils and potential anthropogenic Pb sources are plotted *versus* (^206^Pb/^207^Pb)_a_ per city/village in Figure 4a–f. These figures also include data (plotted as ellipses) for potential anthropogenic sources as reported in other studies (e.g., gasoline Pb, incinerator ash and Dutch and Belgian coal and galena ore). Belgian coal, Dutch coal and gasoline Pb are reported in Walraven *et al.* [14], Walraven *et al.* [19] and Walraven *et al.* [6] respectively. The Pb isotope compositions of Dutch and Belgian galena and incinerator ash in North-Western Europe were determined and published by others [15,16,54,55,56,57,58].

#### 5.1.1. Differences and Similarities between Cities/Villages

Figure 4a–f shows that the Pb isotope compositions of anthropogenic Pb in the majority of the soils (~75%) match that of anthropogenic Pb sources found in these soils. The matching anthropogenic Pb sources are glazed potsherds, glazed roof tiles, building remnants, metal slag, Pb-based paint, Pb sheets, coal ash and other Pb containing artefacts (Table 3). It also matches with coal and galena mined in The Netherlands and Belgium (Figure 4a–f; see Section 5.2 for further discussion). The anthropogenic Pb sources most likely entered the urban soils due to historical smelting activities (production remnants), renovation and demolition of houses (paint flakes, pieces of glazed roof tiles and Pb sheets), disposal of coal ashes from coal stoves in backyards and raising and fertilization of the land. Due to oxidation of peat in Dutch subsoils, land slowly subsides. City waste and manure was used in the past to raise this subsiding land and to fill channels and ditches [14].

The Pb isotope composition of the anthropogenic Pb fraction in ~25% of the urban soils does not match with the inferred potential Pb sources (Figure 4a–f). These samples mainly represent topsoils with Pb isotope compositions matching that of incinerator ash and in some cases gasoline Pb (Figure 4a,c–f). These topsoils might be polluted with atmospheric Pb instead of building materials and household utensils, coal ashes and metal slag. Three soil samples from a historical landfill (L) in Wijk bij Duurstede have anthropogenic Pb isotope compositions that match incinerator ash (Figure 4d). This landfill contains household garbage. The Pb isotope composition of the landfill soil samples most likely matches that of incinerator ash, because household garbage forms an important part of incinerator ash. The Pb isotope composition of several (*n* = 8) topsoils from Fijnaart does not match the anthropogenic Pb sources found in the Fijnaart soils (Figure 4e) but are comparable to sherd (artefact 31; Figure 4c) and glazed potsherd (artefact 47; Figure 4d) found in Wijk bij Duurstede soils. In addition, they have compositions close to the atmospheric Pb sources incinerator ash and gasoline Pb, but the ^207^Pb values are enriched. Since Fijnaart is situated in a highly industrialized area, the topsoils are most likely polluted with an atmospheric Pb source but the exact source is unknown. Four subsoil samples from Fijnaart (S) have enriched Pb contents (Supplementary 1; EF > 2) but their Pb isotope compositions correspond with unpolluted Dutch subsoils (Figure 4e). No potential anthropogenic Pb sources are observed in these soils and it appears that the subsoils are naturally enriched in Pb.

With the exception of the unknown anthropogenic Pb source in some topsoils in Fijnaart, the anthropogenic Pb sources in the soils and cities with a long habitation history are comparable. Due to the high Pb content in glazed potsherds, glazed roof tiles, paint and Pb sheets, these are the sources that influence the anthropogenic Pb content the most. Soils with a large proportion of building materials and household utensils, like in Graft-De Rijp due to the large fires, show the highest Pb contents, up to ~5000 mg/kg (Supplementary 1).

The Pb content and Pb isotope composition of Dutch urban soils in regions with long habitation differs from Dutch rural soils. The median anthropogenic Pb content in the urban soils (140 mg/kg, Table 2) is a factor 10 higher than in the rural soils (13 mg/kg, Walraven *et al.* [19]). The higher anthropogenic Pb content in urban soils is caused by the presence of anthropogenic Pb sources like glazed potsherd and Pb paint flakes that can contain several wt.% Pb (Table 3). Even small pieces of these artefacts can increase the soil Pb content significantly. The median Pb isotope composition of the urban soils also differs from rural soils. The mean (^206^Pb/^207^Pb)_a_, (^208^Pb/^207^Pb)_a_ and (^206^Pb/^208^Pb)_a_ values of the urban soils are 1.171, 2.447 and 0.478 respectively, whereas in rural soils these values are 1.159, 2.441 and 0.475 respectively. Both urban and rural soils contain atmospheric Pb. The observed difference in the anthropogenic Pb isotope composition is caused by the presence of Pb containing artefacts in the urban soils. Figure 4a–f show that the origin of Pb in the artefacts most likely has a more local origin (Germany and Belgium) compared with Pb in atmospheric deposition that can contain Pb imported from Australia (e.g., gasoline Pb).

#### 5.1.2. Differences and Similarities between Historical Time Periods

The anthropogenic Pb content of the Utrecht and Wijk bij Duurstede soils—influenced in the Roman period—is relatively low (<LOD to 15 mg/kg; Supplementary 1). Since the EF of these soils is <2, the Pb isotope composition of anthropogenic Pb in these soils has not been calculated (see Section 3.4). Nevertheless, the Pb isotope compositions of the potential Pb sources from the Roman period in Wijk bij Duurstede are presented in Figure 4b. The Pb isotope composition of the majority (~85%) of Roman potential Pb sources corresponds with the Pb isotope composition of unpolluted Dutch subsoils being derived predominantly from unglazed potsherds most likely made from local clay. The glazed potsherds (Figure 4b: sample 17 and 18) and glass fragment (Figure 4b: sample 22) from the Roman period, found in the Wijk bij Duurstede soils, have Pb isotope compositions that match coal and galena Pb (Figure 4b) and probably contain Pb from Dutch or Belgian Pb ores (galena).

There is no clear difference in Pb isotope composition between Utrecht soils influenced in the Medieval and Modern period (Figure 4a). The Pb isotope compositions of anthropogenic Pb in Wijk bij Duurstede soils influenced during the Medieval and Modern period, with the exception of 1 sample, are also consistent (Figure 4c,d). Based on Pb isotope ratios alone the time periods are essentially indistinguishable. However, the anthropogenic Pb content in the soils influenced during the various time periods does differ (Figure 3). Due to the increased use of lead in a variety of products over time and the increased population in The Netherlands, Pb pollution in urban soils also increased with time [31,36]. Since: (1) there are now strict regulations with respect to raising of land with city waste; (2) coal is no longer used as a primary energy source in The Netherlands and (3) public awareness of risks related to Pb increased significantly in recent years, further increase in the anthropogenic Pb content of city soils is expected to be limited. Careless renovation of (mainly old) houses, however, can still result in anthropogenic Pb (Pb based paint, Pb glazed roof tiles and Pb sheets) entering urban soils (backyards). For this reason, the removal of Pb containing building materials should be carried out with the greatest care, to minimize Pb exposure to children who may play in these yards.

### 5.2. Oral Pb Bioaccessibility 

**Figure 5:**
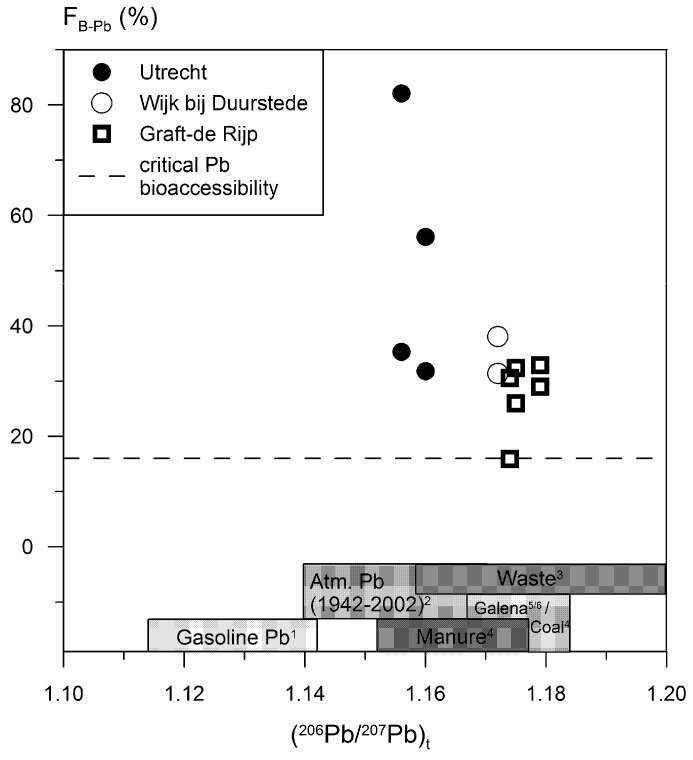
F_B-Pb_ *versus* (^206^Pb/^207^Pb)_t_ of soils from cities and villages with a long habitation history. ^1^ Walraven *et al.* [6]; ^2^ Walraven *et al.* [11]; ^3^ Walraven *et al.* [14] & this study; ^4^ Walraven *et al.* [19]; ^5^ Pasteels *et al.* [54]; ^6^ Cauet *et al.* [55].

In Figure 5 calculated oral bioaccessibilities are plotted *versus* the (^206^Pb/^207^Pb)_t_ ratios of the soil samples (Pb isotope data are lacking for sample U3 and U4). This figure also includes Pb isotope data of known anthropogenic Pb sources in The Netherlands. Table 4 and Figure 5 show that Pb bioaccessibilities for the studied soils decrease in the following order: Utrecht (32%–82%) > Wijk bij Duurstede (31%–38%) ≈ Graft-De Rijp (16%–38%).

The observed difference in oral Pb bioaccessibilities is also reflected in the soil (^206^Pb/^207^Pb)_t_ ratios (Figure 5). The Pb polluted soils from Wijk bij Duurstede and Graft-De Rijp have very distinct (^206^Pb/^207^Pb)_t_ ratios (1.172–1.179) that match with coal/galena and household waste that includes Pb-containing artefacts (Figure 5). Both coal and Pb containing artefacts such as remnants of Pb glazed pottery and roof tiles were visible in these soil samples. In addition, Hagens *et al.* [34] investigated sample GdR3 with a Scanning Electron Microscope (SEM). They concluded that this soil sample is mainly polluted with lead glass and lead glaze—primary Pb phases—with relatively large diameters (up to 675 um). Very few secondary Pb phases (Pb-apatite) were observed and there was a very low Pb content (0.02 wt.%) in the organic matter rich particles in this sample. Hagens *et al.* [34] concluded that the solubility of these primary Pb phases is relatively low, due to the small reactive surface and the incorporation of Pb in a glass matrix. This low solubility most likely resulted in the formation of very few secondary Pb phases. The coarse grain size and relatively insoluble primary Pb phases (coal and glazed potsherds) most likely explains the relatively low oral Pb bioaccessibility of the soil samples from Graft-De Rijp and Wijk bij Duurstede.

The (^206^Pb/^207^Pb)_t_ ratios (1.156–1.160) of the Pb polluted soil samples from Utrecht differ from those of Wijk bij Duurstede and Graft-De Rijp (Figure 5). These ratios match with household waste and atmospheric Pb. In sample U3 to U6 anthropogenic Pb sources were not visible. Sample U5 was analysed with a SEM by Hagens *et al.* [34]. This sample is polluted with very fine primary Pb phases (<1 um). These particles were observed in the elemental map, but could not be detected using energy dispersive X-Ray fluorescence analysis. Based on the presence of the secondary minerals Pb apatite (10–30 um), and Pb containing organic matter (0.160 wt.%) and Fe phases (5–10 um), this soil most likely contains very fine soluble primary Pb phases like native Pb, Pb oxide or Pb carbonate particles [34]. Soil samples U1 and U2 were taken within ~50 m of a former Pb white factory in Utrecht. These samples most likely contain fine grained Pb white particles emitted from the factory. The fine grain size and relatively soluble primary Pb phases (e.g., Pb white) most likely explain the relatively high oral Pb bioaccessibility of the soil samples from Utrecht.

Residence in the soil is also expected to be an important factor. Assuming that oral bioaccessibility eventually decreases with time (soluble primary Pb phases are leached out or are converted to less soluble secondary Pb phases), soils of Graft-De Rijp are expected to have lower bioaccessibilities than soil U1 and U2 from Utrecht. Graft-De Rijp soils were mainly polluted in the 17th century (see Section 2.4), whereas Utrecht U1 and U2 were mainly polluted in the 19th century when the Pb white factory was in operation. Table 4 and Figure 5 show that bioaccessibilities in Graft-De Rijp soils are indeed lower.

Previous workers also determined oral Pb bioaccessibility of Pb polluted soils [32,59,60,61,62,63,64]. Comparison studies have shown that the bioaccessibility methodologies used in these studies differ substantially [52,65]. In addition, in most other studies, anthropogenic Pb sources were not identified or other Pb sources were involved (e.g., shooting ranges, incinerators, landfills, mining and smelting impacted soils) and therefore a comparison with the Dutch urban soils cannot be made.

**Figure 6:**
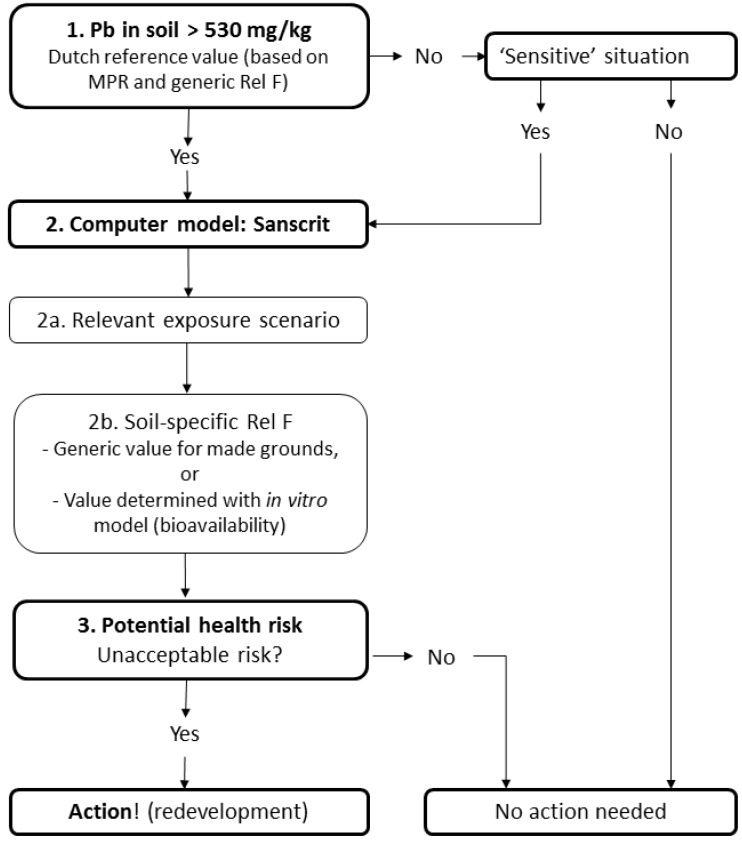
Current practice of risk assessment of Pb in The Netherlands (source: Van Kesteren *et al.* [66]; with permission).

The findings of this study can be used to assess human risk. The current practice of risk assessment of Pb in soils in The Netherlands is illustrated in Figure 6.

The total soil Pb content is measured to determine if a soil is polluted. A soil with a Pb content exceeding the intervention value of 530 mg/kg for standard soils (25% clay and 10% organic matter) is classified as “seriously” contaminated [67]. When this intervention value is not exceeded, no further action is required unless there is a specific “sensitive” situation, such as a vegetable garden (Figure 6). In case of a serious soil contamination (Pb_standard soil_ > 530 mg/kg), the contaminated site has, in principle, to be remediated. The need for remediation, however, is decided on the basis of actual risks to humans and ecosystems and the actual risk due to migration of the contamination. This risk assessment is performed in The Netherlands with the decision-support tool Sanscrit. Two main aspects of the tool can be distinguished (Van Kesteren *et al.* [66]: (1) the relevant human exposure scenario can be calculated and (2) soil-specific evaluation can be performed by determining the relative bioavailability factor (Rel F). This factor is introduced to compare oral bioavailability of Pb in soils with bioavailability of Pb based on toxicity studies with food, liquids and suspensions on which “legal” threshold values are based (see Walraven *et al.* [32] for more details). Rel F (= F_relative_) is calculated according to Equation (5):

F_relative_ = F_soil_ / F_tox.studies_ = (F_B-soil_ × F_A-soil_ × F_H-soil_) / (F_B- tox.studies_ × F_A-tox.studies_ × F_H- tox.studies_) (5)

In which F_relative_ is the relative bioavailability of Pb, F_soil_ is the bioavailability of Pb in soils, F_B-soil_ is the bioaccessible Pb fraction in soil (the fraction that is mobilized from soil into the digestive juice, *i.e.*, chyme), F_A-soil_ is the bioaccessible Pb fraction from the soil entering the portal vein or lymph, F_H-soil_ is the Pb fraction that entered the portal vein or the lymph and passes through the liver without being metabolized (this may exert toxicity in organs and tissues), F_tox.studies_ is the bioavailability of Pb determined in toxicity studies (mainly matrices such as foods and liquids), F_B-tox.studies_ is the bioaccessible Pb fraction determined in toxicity studies, F_A-tox.studies_ is the bioaccessible Pb fraction entering the portal vein or lymph determined in toxicity studies and F_H-tox.studies_ is the Pb fraction that entered the portal vein or the lymph and passes through the liver without being metabolized, determined in toxicity studies.

As a worst case scenario, F_A-soil_ is assumed to be 1 for children. Since inorganic Pb is not metabolized in the liver (ATSDR [68]), F_H_ in both soil and toxicity studies is 1. Based on an absorption of 40% dietary lead (Oomen *et al.* [33]), F_B-tox.studies_ × F_A-tox.studies_ is set at 0.4. Based on these values, Equation (5) becomes:

F_relative_ = (F_B-soil_ × 1 × 1) / (0.4 × 1) = F_B-soil_ / 0.4 (6)

The investigated soils in the four towns are all considered made grounds. For made grounds, Rel F is set to 0.4 in the decision-support tool Sanscrit, implying that F_B-soil_ is 16%. The implication is that if the actual F_B-soil_ is lower than 16%, the bioavailability of Pb in the polluted soils is overestimated by Sanscrit, and vice versa. Table 4 shows that 13 out of 14 soils have bioaccessibilities higher than 16%. This implies that the risk for children may be underestimated at these locations. It is noted, however, that our model represents a worst case scenario. Experiments and calculations are based on fasted conditions. Children are normally fed throughout the day (especially when they play outside).

This finding is comparable to the results of Van Kesteren *et al.* [66] who performed a validation study in which the bioavailability of lead in 6 soils (including soils from Utrecht and Graft-de Rijp) was estimated using three *in vitro* bioavailability models (including the RIVM model). These results were compared with the results of a bioavailability study conducted on juvenile swine (*in vivo* study). The behaviour of lead in the gastrointestinal tract of swine is assumed to be comparable to that in children. Based on the results of their validation study, Van Kesteren *et al.* [66] proposed to increase Rel F to a value in the range from 0.58 (P50) to 0.84 (P80), taking into account the desired level of conservatism.

## 6. Conclusions

Anthropogenic Pb content and isotopic composition were determined on urban soils with a long habitation history. The anthropogenic Pb content of the urban soils varied between < LOD to 5266 mg/kg. The median anthropogenic Pb content increased in the following order: Wijk bij Duurstede (57 mg/kg) < Utrecht (127 mg/kg) < Fijnaart (133 mg/kg) < Graft-De Rijp (789 mg/kg). The median anthropogenic Pb content for the soils from the three distinguished time periods increased in the following order: Roman period (7 mg/kg) < Medieval period (43 mg/kg) < Modern period (235 mg/kg).

The Pb isotope composition of the anthropogenic Pb fraction in the urban soils varied from 1.111 to 1.199, 2.367 to 2.476 and 0.464 to 0.490 for ^206^Pb/^207^Pb)_a_, (^208^Pb/^207^Pb)_a_ and (^206^b/^208^Pb)_a_ respectively. The calculated Pb isotope compositions of anthropogenic Pb in the majority (~75%) of the urban soils appear to represent a mixture of potential anthropogenic Pb sources found in these soils: glazed potsherds, glazed roof tiles, building remnants, metal slag, Pb-based paint, Pb sheets, coal ash and other Pb containing artefacts. These anthropogenic Pb sources most likely entered the urban soils due to historical smelting activities, renovation and demolition of houses, disposal of coal ashes and raising and fertilization of land with city waste. The Pb isotope composition of the anthropogenic Pb fraction in ~25% of the urban soils is inconsistent with the encountered potential Pb sources. These topsoils have Pb isotope compositions consistent with an origin from incinerator ash and in some cases gasoline Pb.

The oral Pb bioaccessibility (F_B-Pb_)—determined with an *in vitro* test performed on soils from villages and cities with a long habitation history—varied from 16% to 82%. F_B-Pb_ appears to be related to the chemical composition and grain size of the primary anthropogenic Pb phases and to pollution age. The smaller the grain size, the more soluble the primary Pb phases and a shorter pollution soil residence time yields a higher oral Pb bioaccessibility. Risk assessment based on the *in vitro* test results (fasted conditions; Rel F is 0.4) shows that in ~90% of the studied samples (13 out of 14) the risk of Pb polluted soil to children may be underestimated.

## Figures and Tables

**Table 1:** Sample locations, site coordinates (RD coordinates), sampling dates and founding dates of the cities and villages.

Location	Abbreviation	Coordinates	Founded in (year)	Sampling Date
Utrecht	U	136,800, 455,900	47 B.C.	October 2000
Wijk bij Duurstede	WbD	151,900, 442,950	57 B.C.	May 2003
Fijnaart	F	91,400, 405,650	1548 A.D.	April 2001
Graft-De Rijp	GdR	118,200, 507,650	1612 A.D.	January 1996

**Table 2:** Summary of the analytical and calculated results of the soils from Utrecht, Wijk bij Duurstede, Fijnaart and Graft-De Rijp.

Measured Parameter	Statistic	All	Utrecht					Wijk bij Duurstede					Fijnaart			Graft-De Rijp		
			All	B	R	Me	Mo	All	B	R	Me	Mo	All	B	Mo	All	B	Mo
Pb_a_ (mg/kg)	n	137	22	2	2	5	13	40	6	5	10	19	30	5	25	45	5	40
	MIN	<LOD	<LOD	<LOD	12	31	51	<LOD	<LOD	<LOD	6	20	<LOD	<LOD	9	<LOD	<LOD	5
	MED	140	127	2	13	67	158	57	3	4	22	94	133	3	225	789	3	790
	MAX	5266	390	2	15	390	387	740	4	7	142	740	1093	3	1093	5266	3	5266
(^206^Pb/^207^Pb)_a_	n	102	18	-	-	5	13	24	-	-	6	18	23	-	23	37	-	37
	MIN	1.111	1.153	-	-	1.167	1.153	1.137	-	-	1.137	1.153	1.130	-	1.130	1.111	-	1.111
	MED	1.171	1.174	-	-	1.169	1.175	1.170	-	-	1.170	1.170	1.163	-	1.163	1.172	-	1.172
	MAX	1.199	1.191	-	-	1.177	1.191	1.180	-	-	1.175	1.180	1.199	-	1.199	1.179	-	1.179
(^208^Pb/^207^Pb)_a_	n	102	18	-	-	5	13	24	-	-	6	18	23	-	23	37	-	37
	MIN	2.367	2.430	-	-	2.431	2.430	2.418	-	-	2.418	2.434	2.367	-	2.367	2.386	-	2.386
	MED	2.447	2.442	-	-	2.447	2.441	2.452	-	-	2.457	2.451	2.432	-	2.432	2.448	-	2.448
	MAX	2.476	2.475	-	-	2.475	2.466	2.459	-	-	2.459	2.459	2.476	-	2.476	2.458	-	2.458
(^206^Pb/^208^Pb)_a_	n	102	18	-	-	5	13	24	-	-	6	18	23	-	23	37	-	37
	MIN	0.465	0.472	-	-	0.474	0.472	0.470	-	-	0.470	0.473	0.472	-	0.472	0.465	-	0.465
	MED	0.478	0.479	-	-	0.478	0.480	0.477	-	-	0.477	0.478	0.479	-	0.479	0.478	-	0.478
	MAX	0.490	0.488	-	-	0.481	0.488	0.480	-	-	0.478	0.480	0.490	-	0.490	0.482	-	0.482
Al (wt.%)	n	137	22	2	2	5	13	40	6	5	10	19	30	5	25	45	5	40
	MIN	0.159	1.988	2.144	4.805	3.004	1.988	2.529	3.884	5.350	2.975	2.529	0.338	0.338	1.006	0.159	3.387	0.159
	MED	3.472	3.273	4.123	4.811	3.910	3.036	4.740	6.367	6.110	4.800	3.610	3.014	3.115	3.013	3.546	6.351	3.440
	MAX	7.357	6.101	6.101	4.817	4.523	3.775	6.997	6.997	6.292	5.842	6.072	4.147	3.683	4.147	7.357	7.357	7.251

MIN = minimum; MED = median; MAX = maximum; LOD = limit of detection; R = Roman Period; Me = Medieval Period; Mo = Modern Period; B = background.

**Table 3:** Analytical results of the (potential) anthropogenic Pb sources (Pb artefacts).

Sample	Nr.	Site	Period	Century	Al (wt.%)	Pb (mg/kg)	^206^Pb/^207^Pb	^208^Pb/^207^Pb	^206^Pb/^208^Pb
potsherd	1	U	Me	-	N.D.	47	1.187	2.496	0.476
glazed roof tile	2	U	Mo	-	5.963	4948	1.188	2.450	0.485
building remnants	3	U	Mo	-	3.594	513	1.180	2.436	0.484
metal slag	4	U	Mo	-	3.550	106	1.156	2.447	0.473
potsherd	5	WbD	R	-	7.993	26	1.204	2.472	0.487
potsherd	6	WbD	R	-	7.546	28	1.205	2.475	0.487
potsherd	7	WbD	R	-	11.432	35	1.203	2.476	0.486
potsherd	8	WbD	R	-	10.013	32	1.206	2.480	0.486
potsherd	9	WbD	R	-	6.803	21	1.195	2.474	0.483
potsherd	10	WbD	R	-	9.785	31	1.200	2.480	0.484
potsherd	11	WbD	R	-	11.436	24	1.197	2.479	0.483
potsherd	12	WbD	R	-	10.061	33	1.204	2.478	0.486
potsherd	13	WbD	R	-	8.487	28	1.204	2.477	0.486
potsherd	14	WbD	R	-	12.382	39	1.206	2.480	0.486
potsherd	15	WbD	R	-	8.989	11	1.195	2.476	0.483
potsherd	16	WbD	R	-	12.259	40	1.200	2.476	0.485
glazed potsherd	17	WbD	R	-	7.862	13129	1.182	2.458	0.481
glazed potsherd	18	WbD	R	-	6.572	26836	1.173	2.449	0.479
sherd	19	WbD	R	mid	8.705	297	1.185	2.460	0.482
sherd	20	WbD	R	mid	9.564	194	1.199	2.472	0.485
sherd	21	WbD	R	early	7.413	156	1.204	2.474	0.487
glass	22	WbD	R	-	1.306	8	1.170	2.444	0.479
sherd	23	WbD	R	3/4	6.202	22	1.199	2.456	0.488
production slag	24	WbD	Me	-	1.195	12	1.195	2.467	0.485
production slag	25	WbD	Me	-	6.871	29	1.203	2.476	0.486
production slag	26	WbD	Me	-	1.583	3	1.196	2.468	0.484
sherd	27	WbD	Me	-	8.175	23	1.204	2.490	0.484
sherd	28	WbD	Me	-	9.833	27	1.205	2.486	0.485
sherd	29	WbD	Me	-	14.704	51	1.190	2.474	0.481
glass	30	WbD	Me	early	1.147	6103	1.207	2.484	0.486
sherd	31	WbD	Me	car.	8.062	38	1.150	2.405	0.478
sherd	32	WbD	Me	car.	7.514	27	1.182	2.462	0.480
glazed potsherd	33	WbD	Me	10/11	10.444	31138	1.177	2.457	0.479
glazed potsherd	34	WbD	Me	11	10.421	12620	1.177	2.458	0.479
glazed potsherd	35	WbD	Me	12	10.378	5345	1.177	2.456	0.479
glazed potsherd	36	WbD	Me	13	8.189	30529	1.178	2.458	0.479
glazed potsherd	37	WbD	Me	14	7.291	9760	1.181	2.460	0.480
prod. remnants	38	WbD	Me	-	5.208	58	1.182	2.466	0.480
prod. remnants	39	WbD	Me	-	4.595	712	1.179	2.458	0.480
prod. remnants	40	WbD	Me	-	3.854	66	1.178	2.458	0.479
prod. remnants	41	WbD	Me	-	0.143	651	1.179	2.456	0.480
prod. remnants	42	WbD	Me	-	1.070	2265	1.165	2.442	0.477
glazed potsherd	43	WbD	Mo	-	6.688	37038	1.174	2.457	0.478
glazed potsherd	44	WbD	Mo	-	5.682	16868	1.175	2.454	0.479
glazed potsherd	45	WbD	Mo	16/17	11.404	10049	1.183	2.463	0.480
glazed potsherd	46	WbD	Mo	16	7.071	40994	1.184	2.460	0.481
glazed potsherd	47	WbD	Mo	17	7.274	15354	1.152	2.397	0.480
glazed potsherd	48	WbD	Mo	17	6.978	86904	1.171	2.451	0.478
glazed potsherd	49	WbD	Mo	17	6.227	16845	1.180	2.455	0.481
majolica	50	WbD	Mo	17	4.349	19478	1.185	2.458	0.482
glazed tile	51	WbD	Mo	17	5.139	11544	1.186	2.461	0.482
glazed potsherd	52	WbD	Mo	17	7.551	21518	1.185	2.459	0.482
glazed potsherd	53	WbD	Mo	18	7.399	6667	1.178	2.452	0.481
Pb spool	54	WbD	Mo	-	0.007	878370	1.179	2.453	0.481
potsherd	55	F	Mo	-	N.D.	39	1.181	2.460	0.480
coal ashes	56	F	Mo	-	N.D.	123	1.161	2.446	0.475
glazed rooftile	57	F	Mo	-	N.D.	23106	1.172	2.451	0.478
metal slag	58	F	Mo	-	N.D.	41	1.167	2.452	0.476
coal ashes	59	F	Mo	-	N.D.	2182	1.174	2.447	0.480
glazed roof tile	60	F	Mo	-	N.D.	19300	1.176	2.452	0.479
metal slag	61	F	Mo	-	N.D.	82	1.170	2.445	0.479
metal slag	62	F	Mo	-	N.D.	1502	1.159	2.442	0.474
undefined object	63	GdR	Mo	-	N.D.	196	1.168	2.448	0.477
glazed red roof tile	64	GdR	Mo	-	6.822	59419	1.176	2.450	0.480
glazed red roof tile	65	GdR	Mo	-	6.843	40186	1.177	2.453	0.480
red roof tile	66	GdR	Mo	-	7.664	166	1.164	2.443	0.476
red roof tile	67	GdR	Mo	-	7.743	189	1.167	2.452	0.476
glazed chimney pot	68	GdR	Mo	-	7.447	27697	1.194	2.478	0.482
grey roof tile	69	GdR	Mo	-	9.918	51	1.183	2.459	0.481
red paint (on wood)	70	GdR	Mo	-	0.090	185665	1.196	2.468	0.485
impregnated wood	71	GdR	Mo	-	0.217	4918	1.192	2.466	0.483
brown paint	72	GdR	Mo	-	N.D.	107896	1.160	2.445	0.474
white paint	73	GdR	Mo	-	0.894	60074	1.171	2.451	0.478
Pb sheet 1	74	GdR	Mo	-	N.D.	~1000000	1.165	2.439	0.478
Pb sheet 2	75	GdR	Mo	-	N.D.	~1000000	1.166	2.464	0.473

century = determined by archaeologists (for all Pb sources the archeological period could be determined, but not always the exact century in which they were made); car. = Carolingian; mid = middle.

**Table 4:** Lead isotope composition of the soil samples used in the *in vitro* digestion model, Pb content of the soils, chyme and pellets, and F_B-Pb_ (Hagens *et al.* [34]) (see Equation (4)).

Location	Sample Name	Inferred Pb Source	Soil			Chyme	Pellet	Bioaccessibility
			Pb (mg/kg)	^206^Pb/^207^Pb	^208^Pb/^207^Pb	Pb (mg/kg)	Pb (mg/kg)	F_B-Pb_ (%)
Utrecht 1	U1	City waste (Pb white)	1443	1.156	2.439	509	484	35
Utrecht 1	U2	City waste (Pb white)	1651			1355	1204	82
Utrecht 2	U3	City waste	1237	-	-	777	789	63
Utrecht 2	U4	City waste	1275			541	581	42
Utrecht 3	U5	City waste	952	1.160	2.437	303	313	32
Utrecht 3	U6	City waste	927			520	577	56
Wijk bij Duurstede 1	WbD1	City waste	639	1.172 *	2.453 *	201	168	31
Wijk bij Duurstede 1	WbD2	City waste	1431			545	490	38
Graft-de Rijp 1	GdR1	City waste	965	1.179 *	2.455 *	317	279	33
Graft-de Rijp 2	GdR2	City waste	2335	1.175	2.456	677	888	29
Graft-de Rijp 2	GdR3	City waste	1947			506	425	26
Graft-de Rijp 3	GdR4	City waste	540	1.174	2.451	175	322	32
Graft-de Rijp 3	GdR5	City waste	1549			474	855	31
Graft-de Rijp 4	GdR6	City waste	1668	1.174 *	2.450 *	265	524	16

* measured in different soil sample, but from the same sample location.

## Supplementary Material

The following is available online at The following are available online at www.mdpi.com/www.mdpi.com/1660-4601/13/2/221/s1, Table S1: Database with the analytical and calculated results of the soil samples.
