# Supplementary material for: Pollution and Oral Bioaccessibility of Pb in Soils of Villages and Cities with a Long Habitation History
Source: Int J Environ Res Public Health. 2016 Feb 17;13(2):221. doi: 10.3390/ijerph13020221 (PMC4772241; doi:10.3390/ijerph13020221)
Supplement: Supplementary file 1 [file ijerph-13-00221-s001.pdf]

Supplementary Material: Pollution and Oral Bioaccessibility of Pb in Soils of Villages and Cities with a Long Habitation History

Nikolaj Walraven, Martine Bakker, Bertil van Os, Gerard Klaver, Jack Middelburg and Gareth Davies

Table S1. Database with the analytical and calculated results of the soil samples.

Location	Sample	Depth (cm–gL)	Lithology	Undisturbed	Pollution Period	Al (wt.%)	Pb (mg/kg)	^{206}Pb/^{207}Pb	^{208}Pb/^{207}Pb	^{206}Pb/^{208}Pb	Pb$_n$ (mg/kg)	Pb$_a$ (mg/kg)	EF	(^{206}Pb/^{207}Pb)$_a$	(^{208}Pb/^{207}Pb)$_a$	(^{206}Pb/^{208}Pb)$_a$
Utrecht	1	0–20	sand	No	Modern	2.597	232	1.180	2.432	0.485	11	221	20.5	1.179	2.430	0.485
Utrecht	2	54–80	sand	No	Modern	2.682	169	1.183	2.435	0.486	12	158	14.5	1.182	2.433	0.486
Utrecht	3	100–120	sand	No	Modern	3.536	114	1.191	2.442	0.488	15	99	7.7	1.191	2.439	0.488
Utrecht	4	0–20	sand	No	Modern	3.106	87	1.175	2.439	0.482	13	74	6.6	1.172	2.435	0.481
Utrecht	5	60–90	sand	No	Modern	3.036	248	1.181	2.434	0.485	13	235	19.1	1.180	2.432	0.485
Utrecht	6	10–40	sand	No	Modern	2.610	201	1.170	2.444	0.479	11	189	17.6	1.168	2.443	0.478
Utrecht	7	60–80	sand	No	Modern	2.324	62	1.175	2.445	0.480	10	51	6.0	1.171	2.441	0.480
Utrecht	8	0–15	fluviatile clay	No	Modern	3.249	166	1.175	2.448	0.480	14	153	12.1	1.173	2.446	0.479
Utrecht	9	50–70	fluviatile clay	No	Medieval	3.805	136	1.179	2.450	0.481	16	120	8.6	1.177	2.447	0.481
Utrecht	10	105–120	fluviatile clay	No	Medieval	4.518	85	1.175	2.440	0.481	18	67	4.6	1.168	2.431	0.481
Utrecht	11	0–60	fluviatile clay	No	Modern	3.297	190	1.183	2.453	0.482	14	176	13.6	1.182	2.451	0.482
Utrecht	12	80–120	fluviatile clay	No	Medieval	3.004	44	1.176	2.451	0.480	13	31	3.4	1.167	2.442	0.478
Utrecht	13	0–10	sand	No	Modern	1.988	147	1.155	2.438	0.474	9	138	16.2	1.153	2.437	0.473
Utrecht	14	60–80	fluviatile clay	No	Modern	3.393	142	1.177	2.455	0.480	14	127	9.9	1.175	2.453	0.479
Utrecht	15	180–195	sand	Yes	Background	2.144	10	1.184	2.458	0.482	10	<LOD	1.0	NC	NC	NC
Utrecht	16	0–10	sand	No	Modern	2.378	335	1.160	2.454	0.473	11	324	31.8	1.159	2.454	0.472
Utrecht	17	50–110	sand	No	Modern	3.775	402	1.175	2.466	0.477	16	387	25.7	1.175	2.466	0.476
Utrecht	18	110–200	fluviatile clay	No	Medieval	3.910	407	1.170	2.464	0.475	16	390	25.1	1.169	2.464	0.474
Utrecht	19	200–350	fluviatile clay	No	Medieval	4.523	62	1.184	2.474	0.478	18	43	3.3	1.177	2.475	0.476
Utrecht	20	350–390	fluviatile clay	No	Roman	4.817	31	1.173	2.470	0.475	20	12	1.6	NC	NC	NC
Utrecht	21	390–400	fluviatile clay	No	Roman	4.805	34	1.179	2.469	0.478	19	15	1.7	NC	NC	NC
Utrecht	22	400–420	fluviatile clay	Yes	Background	6.101	26	1.181	2.465	0.479	24	2	1.1	NC	NC	NC
Fijnaart	1	0–20	marine clay	No	Modern	3.563	387	1.164	2.417	0.482	15	372	26.0	1.163	2.415	0.481
Fijnaart	2	110–135	marine clay	No	Modern	2.681	21	1.193	2.469	0.483	12	9	1.8	NC	NC	NC
Fijnaart	3	0–25	marine clay	No	Modern	3.313	239	1.163	2.414	0.482	14	225	17.1	1.161	2.410	0.482

Fijnaart	4	110–130	marine clay	No	Modern	2.768	26	1.182	2.459	0.481	12	14	2.2	1.165	2.445	0.477
Fijnaart	5	0–25	marine clay	No	Modern	3.015	282	1.159	2.403	0.482	13	269	21.9	1.157	2.400	0.482
Fijnaart	6	100–120	marine clay	No	Modern	3.077	35	1.192	2.474	0.482	13	22	2.7	1.186	2.473	0.480
Fijnaart	7	0–25	marine clay	No	Modern	3.339	464	1.167	2.420	0.482	14	450	33.0	1.166	2.419	0.482
Fijnaart	8	120–140	sand	No	Modern	2.584	21	1.190	2.470	0.482	11	10	1.9	NC	NC	NC
Fijnaart	9	0–25	marine clay	No	Modern	2.682	394	1.155	2.421	0.477	12	383	33.9	1.154	2.420	0.477
Fijnaart	10	130–150	sand	No	Modern	2.845	39	1.197	2.472	0.484	12	27	3.2	1.199	2.476	0.484
Fijnaart	11	0–25	marine clay	No	Modern	3.167	695	1.173	2.413	0.486	13	682	51.8	1.172	2.412	0.486
Fijnaart	12	0–25	marine clay	No	Modern	2.629	400	1.156	2.421	0.477	11	389	35.0	1.155	2.419	0.477
Fijnaart	13	0–25	marine clay	No	Modern	1.951	139	1.147	2.399	0.478	9	130	15.6	1.144	2.393	0.478
Fijnaart	14	80–100	sand	No	Modern	2.751	28	1.186	2.469	0.480	12	17	2.4	1.180	2.473	0.477
Fijnaart	15	0–25	marine clay	No	Modern	2.744	1105	1.160	2.368	0.490	12	1093	93.0	1.159	2.367	0.490
Fijnaart	16	25–50	marine clay	No	Modern	2.051	450	1.169	2.433	0.481	9	441	48.3	1.169	2.432	0.481
Fijnaart	17	110–130	marine clay	No	Modern	3.013	42	1.197	2.476	0.484	13	29	3.2	1.195	2.476	0.483
Fijnaart	18	0–25	marine clay	No	Modern	3.184	363	1.171	2.443	0.479	13	350	26.9	1.170	2.442	0.479
Fijnaart	19	150–170	marine clay	No	Modern	3.855	334	1.175	2.423	0.485	16	318	20.9	1.174	2.420	0.485
Fijnaart	20	0–25	marine clay	No	Modern	2.715	80	1.141	2.408	0.474	12	68	6.8	1.130	2.397	0.472
Fijnaart	21	200–250	marine clay	Yes	Background	3.683	14	1.209	2.492	0.485	15	<LOD	0.9	NC	NC	NC
Fijnaart	22	325–350	peat	Yes	Background	3.115	13	1.206	2.485	0.485	13	<LOD	1.0	NC	NC	NC
Fijnaart	23	410–450	peat	No	Modern	1.006	15	1.170	2.450	0.477	5	10	2.8	1.158	2.438	0.475
Fijnaart	24	0–20	marine clay	No	Modern	3.472	60	1.170	2.457	0.476	15	45	4.1	1.159	2.451	0.473
Fijnaart	25	200–250	marine clay	Yes	Background	3.449	18	1.204	2.484	0.485	14	3	1.2	NC	NC	NC
Fijnaart	26	325–370	peat	Yes	Background	0.977	8	1.186	2.466	0.481	5	3	1.5	NC	NC	NC
Fijnaart	27	370–400	peat	Yes	Background	0.338	4	1.179	2.453	0.481	3	<LOD	1.3	NC	NC	NC
Fijnaart	28	0–50	marine clay	No	Modern	3.754	287	1.160	2.437	0.476	16	272	18.4	1.157	2.435	0.475
Fijnaart	29	80–130	marine clay	No	Modern	4.147	965	1.159	2.436	0.476	17	948	56.6	1.158	2.435	0.476
Fijnaart	30	170–220	marine clay	No	Modern	3.993	149	1.177	2.457	0.479	16	133	9.1	1.174	2.455	0.478
De Rijp	1	12.5–37.5	marine clay	No	Modern	3.440	720	1.173	2.449	0.479	14	706	49.9	1.172	2.449	0.479
De Rijp	2	62.5–87.5	marine clay	No	Modern	5.928	461	1.168	2.440	0.479	24	437	19.5	1.167	2.438	0.478
De Rijp	3	162.5–187.5	peat	No	Modern	0.159	8	1.180	2.455	0.480	2	5	3.2	NC	NC	NC
De Rijp	4	12.5–37.5	marine clay	No	Modern	3.070	745	1.175	2.450	0.480	13	732	56.9	1.175	2.450	0.480
De Rijp	5	185–195	marine clay	Yes	Background	6.616	29	1.202	2.468	0.487	26	3	1.1	NC	NC	NC
De Rijp	6	12.5–37.5	marine clay	No	Modern	3.440	331	1.168	2.441	0.478	14	316	22.9	1.166	2.439	0.478
De Rijp	7	62.5–87.5	marine clay	No	Modern	2.540	515	1.176	2.448	0.480	11	504	46.3	1.176	2.448	0.480

De Rijp	8	112.5–137.5	marine clay	No	Modern	3.017	2001	1.176	2.447	0.481	13	1988	155.3	1.176	2.447	0.481
De Rijp	9	27.5–42.5	peat	No	Modern	4.446	52	1.178	2.455	0.480	18	34	2.9	1.171	2.446	0.479
De Rijp	10	155–185	marine clay	Yes	Background	6.351	23	1.200	2.478	0.484	25	<LOD	0.9	NC	NC	NC
De Rijp	11	12.5–37.5	marine clay	No	Modern	5.875	43	1.186	2.462	0.482	23	19	1.8	NC	NC	NC
De Rijp	12	62.5–87.5	marine clay	No	Modern	5.769	31	1.197	2.471	0.484	23	8	1.3	NC	NC	NC
De Rijp	13	112.5–137.5	marine clay	Yes	Background	3.387	11	1.204	2.492	0.483	14	<LOD	0.8	NC	NC	NC
De Rijp	14	12.5–37.5	marine clay	No	Modern	5.769	46	1.183	2.467	0.480	23	23	2.0	1.164	2.458	0.474
De Rijp	15	120–140	marine clay	Yes	Background	3.705	12	1.207	2.487	0.485	15	<LOD	0.7	NC	NC	NC
De Rijp	16	0–50	marine clay	No	Modern	4.869	1693	1.164	2.438	0.478	20	1674	85.9	1.164	2.437	0.478
De Rijp	17	0–50	sand	No	Modern	3.758	58	1.174	2.453	0.479	16	42	3.7	1.167	2.449	0.476
De Rijp	18	0–50	sand	No	Modern	3.123	615	1.172	2.453	0.478	13	602	46.3	1.172	2.453	0.478
De Rijp	19	0–50	sand	No	Modern	2.593	802	1.152	2.423	0.475	11	791	70.9	1.152	2.423	0.475
De Rijp	20	0–200	marine clay	No	Modern	4.763	1728	1.160	2.438	0.476	19	1708	89.4	1.159	2.437	0.476
De Rijp	21	0–50	sand	No	Modern	2.911	1447	1.179	2.452	0.481	12	1434	115.8	1.178	2.452	0.481
De Rijp	22	0–50	sand	No	Modern	4.499	5284	1.158	2.434	0.476	18	5266	288.0	1.158	2.434	0.476
De Rijp	23	0–200	sand	No	Modern	3.017	1659	1.172	2.451	0.478	13	1646	128.8	1.172	2.450	0.478
De Rijp	24	0–50	sand	No	Modern	3.969	1185	1.173	2.445	0.480	16	1169	72.3	1.173	2.445	0.480
De Rijp	25	0–200	sand	No	Modern	2.911	1093	1.173	2.445	0.480	12	1081	87.5	1.173	2.445	0.480
De Rijp	26	0–200	sand	No	Modern	2.858	1124	1.178	2.449	0.481	12	1111	91.4	1.177	2.449	0.481
De Rijp	27	0–200	sand	No	Modern	2.646	944	1.165	2.439	0.477	12	932	82.0	1.164	2.439	0.477
De Rijp	28	0–50	sand	No	Modern	2.964	1598	1.173	2.455	0.478	13	1586	126.0	1.173	2.455	0.478
De Rijp	29	0–50	sand	No	Modern	2.858	2792	1.111	2.387	0.466	12	2780	227.1	1.111	2.386	0.465
De Rijp	30	0–200	sand	No	Modern	2.911	786	1.180	2.455	0.480	12	774	62.9	1.179	2.455	0.480
De Rijp	31	0–200	sand	No	Modern	3.546	290	1.179	2.445	0.482	15	275	19.6	1.178	2.444	0.482
De Rijp	32	0–50	sand	No	Modern	3.017	1721	1.175	2.450	0.479	13	1708	133.6	1.174	2.450	0.479
De Rijp	33	100–130	marine clay	Yes	Background	7.357	24	1.206	2.474	0.488	29	<LOD	0.8	NC	NC	NC
De Rijp	34	0.5–25.5	marine clay	No	Modern	7.092	90	1.178	2.457	0.479	28	62	3.2	1.167	2.449	0.477
De Rijp	35	0.5–25.5	marine clay	No	Modern	4.340	1311	1.175	2.455	0.479	18	1293	73.8	1.175	2.455	0.478
De Rijp	36	40–60	marine clay	No	Modern	4.499	1128	1.173	2.447	0.479	18	1109	61.5	1.172	2.447	0.479
De Rijp	37	40–100	marine clay	No	Modern	4.393	2087	1.173	2.451	0.478	18	2069	116.2	1.172	2.451	0.478
De Rijp	38	0–40	marine clay	No	Modern	2.699	347	1.165	2.449	0.476	12	335	29.6	1.164	2.448	0.475
De Rijp	39	0–40	sand	No	Modern	3.281	1112	1.167	2.447	0.477	14	1098	80.2	1.167	2.447	0.477
De Rijp	40	0–40	marine clay	No	Modern	4.234	602	1.171	2.448	0.478	17	584	34.6	1.170	2.447	0.478

De Rijp	41	50–110	marine clay	No	Modern	4.234	806	1.174	2.456	0.478	17	789	46.4	1.173	2.455	0.478
De Rijp	42	120–160	marine clay	No	Modern	3.123	1218	1.174	2.452	0.479	13	1204	91.7	1.174	2.452	0.479
De Rijp	43	150–180	marine clay	No	Modern	3.387	1727	1.174	2.456	0.478	14	1712	121.2	1.174	2.456	0.478
De Rijp	44	0.5–25.5	marine clay	No	Modern	6.880	226	1.169	2.453	0.476	27	199	8.3	1.164	2.449	0.475
De Rijp	45	0–30	marine clay	No	Modern	7.251	106	1.171	2.442	0.480	29	77	3.7	1.160	2.429	0.478
Wijk bij Duurstede	1	5–15	fluviatile clay	No	Modern	6.072	77	1.181	2.458	0.481	24	53	3.2	1.173	2.451	0.479
Wijk bij Duurstede	2	30–50	fluviatile clay	No	Roman	6.017	26	1.198	2.471	0.485	24	2	1.1	NC	NC	NC
Wijk bij Duurstede	3	100–110	fluviatile clay	Yes	Background	6.605	20	1.205	2.478	0.486	26	<LOD	0.8	NC	NC	NC
Wijk bij Duurstede	4	0–10	fluviatile clay	No	Modern	5.657	43	1.181	2.458	0.481	23	20	1.9	NC	NC	NC
Wijk bij Duurstede	5	40–60	fluviatile clay	No	Roman	5.350	22	1.193	2.469	0.483	21	<LOD	1.0	NC	NC	NC
Wijk bij Duurstede	6	70–90	fluviatile clay	Yes	Background	4.399	16	1.199	2.478	0.484	18	<LOD	0.9	NC	NC	NC
Wijk bij Duurstede	7	0–10	fluviatile clay	No	Modern	5.815	51	1.178	2.458	0.479	23	28	2.2	1.160	2.446	0.475
Wijk bij Duurstede	8	30–40	fluviatile clay	No	Roman	6.110	31	1.186	2.464	0.481	24	7	1.3	NC	NC	NC
Wijk bij Duurstede	9	50–60	fluviatile clay	Yes	Background	6.997	27	1.199	2.474	0.485	28	<LOD	1.0	NC	NC	NC
Wijk bij Duurstede	10	0–10	fluviatile clay	No	Modern	5.733	102	1.177	2.455	0.479	23	79	4.5	1.171	2.451	0.478
Wijk bij Duurstede	11	40–50	fluviatile clay	No	Roman	6.262	29	1.193	2.470	0.483	25	4	1.2	NC	NC	NC
Wijk bij Duurstede	12	80–90	fluviatile clay	No	Roman	6.292	25	1.197	2.474	0.484	25	<LOD	1.0	NC	NC	NC
Wijk bij Duurstede	13	100–110	fluviatile clay	Yes	Background	6.129	29	1.195	2.472	0.484	24	4	1.2	NC	NC	NC
Wijk bij Duurstede	14	0–10	fluviatile clay	No	Modern	4.859	46	1.179	2.458	0.480	20	26	2.3	1.164	2.447	0.476
Wijk bij Duurstede	15	50–60	fluviatile clay	No	Medieval	5.067	162	1.145	2.425	0.472	20	142	7.9	1.137	2.418	0.470
Wijk bij Duurstede	16	80–90	fluviatile clay	No	Medieval	5.147	121	1.178	2.460	0.479	21	100	5.8	1.174	2.457	0.478
Wijk bij Duurstede	17	110–120	fluviatile clay	Yes	Background	6.894	29	1.193	2.471	0.483	27	2	1.1	NC	NC	NC
Wijk bij Duurstede	18	60–80	fluviatile clay	No	Medieval	4.852	124	1.179	2.461	0.479	20	104	6.3	1.175	2.459	0.478

Wijk bij Duurstede	19	100–120	fluviatile clay	No	Medieval	5.842	47	1.181	2.464	0.480	23	24	2.0	1.164	2.455	0.475
Wijk bij Duurstede	20	20–30	fluviatile clay	No	Modern	3.761	139	1.171	2.455	0.477	16	124	8.9	1.168	2.452	0.476
Wijk bij Duurstede	21	50–60	fluviatile clay	No	Medieval	4.254	75	1.176	2.460	0.478	17	57	4.3	1.169	2.456	0.476
Wijk bij Duurstede	22	20–30	fluviatile clay	No	Modern	4.393	98	1.171	2.454	0.477	18	80	5.4	1.165	2.450	0.476
Wijk bij Duurstede	23	40–60	fluviatile clay	No	Medieval	4.748	38	1.182	2.463	0.480	19	19	2.0	NC	NC	NC
Wijk bij Duurstede	24	70–80	fluviatile clay	No	Medieval	3.972	37	1.183	2.464	0.480	16	21	2.3	1.171	2.457	0.477
Wijk bij Duurstede	25	95–100	fluviatile clay	No	Medieval	2.975	21	1.183	2.466	0.480	13	8	1.6	NC	NC	NC
Wijk bij Duurstede	26	100–130	fluviatile clay	No	Medieval	5.100	27	1.191	2.470	0.482	21	6	1.3	NC	NC	NC
Wijk bij Duurstede	27	70–80	fluviatile clay	No	Modern	4.689	69	1.175	2.457	0.478	19	50	3.6	1.166	2.452	0.476
Wijk bij Duurstede	28	100–110	fluviatile clay	No	Medieval	4.732	33	1.184	2.465	0.480	19	14	1.7	NC	NC	NC
Wijk bij Duurstede	29	130–140	fluviatile clay	Yes	Background	3.884	19	1.191	2.471	0.482	16	3	1.2	NC	NC	NC
Wijk bij Duurstede	30	0–30	fluviatile clay	No	Modern	2.625	205	1.173	2.454	0.478	11	193	17.9	1.172	2.453	0.478
Wijk bij Duurstede	31	30–60	fluviatile clay	No	Modern	2.529	213	1.170	2.449	0.478	11	202	19.2	1.168	2.448	0.477
Wijk bij Duurstede	32	60–90	fluviatile clay	No	Modern	2.868	221	1.178	2.457	0.479	12	209	17.9	1.177	2.457	0.479
Wijk bij Duurstede	33	90–120	fluviatile clay	No	Modern	3.060	228	1.181	2.460	0.480	13	215	17.5	1.180	2.459	0.480
Wijk bij Duurstede	34	120–150	fluviatile clay	No	Modern	3.254	108	1.182	2.459	0.481	14	94	7.8	1.179	2.457	0.480
Wijk bij Duurstede	35	20–50	fluviatile clay	No	Modern	3.610	373	1.176	2.453	0.479	15	357	24.7	1.175	2.452	0.479
Wijk bij Duurstede	36	60–80	fluviatile clay	No	Modern	3.970	94	1.181	2.460	0.480	16	77	5.7	1.177	2.458	0.479
Wijk bij Duurstede	37	0–30	fluviatile clay	No	Modern	3.358	88	1.160	2.446	0.475	14	73	6.2	1.153	2.441	0.473
Wijk bij Duurstede	38	60–90	fluviatile clay	No	Modern	3.402	298	1.157	2.437	0.475	14	284	20.8	1.155	2.436	0.474
Wijk bij Duurstede	39	100–120	fluviatile clay	No	Modern	2.988	752	1.160	2.435	0.476	13	740	58.9	1.159	2.434	0.476

Wijk bij Duurstede	40	0–5	fluviatile clay	No	Modern	3.322	148	1.179	2.453	0.481	14	134	10.6	1.177	2.451	0.480

NC = not calculated.

© 2016 by the authors; licensee MDPI, Basel, Switzerland. This article is an open access article distributed under the terms and conditions of the Creative Commons by Attribution (CC–BY) license (http://creativecommons.org/licenses/by/4.0/).
